# The relationship between family diet consumption, family environment, parent anxiety and nutrition status children during the COVID-19 pandemic: a longitudinal study

**DOI:** 10.3389/fpubh.2023.1228626

**Published:** 2023-08-10

**Authors:** Lili Peng, Rui Hu, Yiwei Feng, Wei Shi, Li Zhao, Lihua Jiang

**Affiliations:** ^1^Department of Health Policy and Management, West China School of Public Health and West China Fourth Hospital, Sichuan University, Chengdu, China; ^2^Department of Natural Science, Duke Kunshan University, Kunshan, China; ^3^Institute for Disaster Management and Reconstruction (IDMR), Sichuan University, Chengdu, China; ^4^Institute for Hospital Management, West China Hospital, Sichuan University, Chengdu, China

**Keywords:** children’s nutrition status, family diet consumption, family environment, parent anxiety, COVID-19

## Abstract

**Background:**

The COVID-19 pandemic has exacerbated the trends of childhood overweight, obesity, and malnutrition, as well as increased psychological stress and family conflicts among family members. It is important to explore the relationship between changes in the family environment during the COVID-19 on child nutrition.

**Objective:**

This study aims to analyze the nutritional status of Chinese children during the COVID-19 pandemic and its relationship with family diet, family environment, and parental anxiety, in order to provide evidence for further interventions in children’s nutritional status.

**Method:**

This study included 7,645 primary and secondary school students and their parents from five schools in Chengdu, China. Chi-square tests were used to analyze the categorical variables of children’s malnutrition, overweight, obesity, and parental anxiety. T-tests were used to assess changes in the continuous variable of family environment between two rounds of follow-up surveys. Multiple logistic regression analysis was employed to examine the impact of changes in family diet during the COVID-19 pandemic on children’s nutritional status. Generalized estimating equations were used to analyze the effects of family environment and parental anxiety on childhood obesity.

**Result:**

The prevalence of malnutrition and obesity decreased from 11.64% and 11.60% in wave 1 to 4.96% and 10.50% in wave 2, and the rate of overweight increased from 13.11% in wave 1 to 13.73% in wave 2. Children whose families reduced consumption of staple foods during the COVID-19 were more likely to be frail, and families increased consumption of sugary drinks, take-out or meal delivery services, living in towns, family environmental barriers, and parental anxiety were risk factors for overweight obesity. Mother’s education level in middle and high school and low age were protective factors for overweight obesity.

**Conclusion:**

The physical environment of the family, the emotions of family members, and children’s perceptions of the family’s soft environment can influence children’s eating behaviors, children’s nutritional intake, and malnutrition and obesity in children under public health emergencies, and family-based dietary interventions may be effective. Parents can increase consumption of healthy foods and improve the family environment, which improve their growth.

## 1. Introduction

During the COVID-19 pandemic, there has been a simultaneous presence of childhood overweight and obesity as well as malnutrition, with the pandemic response measures exacerbating this challenge (1, 2). According to the World Health Organization, nearly 39 million children were overweight in 2020, and there were almost 340 million overweight or obese children aged 5 to 18 years (3). Additionally, over 149 million children under the age of five experienced stunted growth, and in 2021, the global population affected by hunger reached 828 million (4). The school-age period is a nutritionally sensitive period for growth, and inadequate or excessive nutrient intake can impact children’s cognitive and physical development, as well as their long-term health in adulthood (5, 6). Good nutrition is beneficial for well-being throughout the entire lifespan (1, 7, 8).

The outbreak of the COVID-19 pandemic has brought challenges to the stability of the family environment and the emotions of family members. According to reports, during the outbreak in China, about 25 million rural migrant workers were unemployed or waiting to return to work, and nearly one-fourth of the urban employed population was economically affected (9, 10). Financial difficulties caused by the pandemic have forced many families to make cheaper and unhealthy food choices when purchasing food. Poverty and low-income families may not be able to access sufficient nutrition and food (11, 12). The “lockdown” or “semi-lockdown” situation has caused instability in the family environment, increased parental stress, increased family conflicts, and impaired family functioning, severely affecting the children’s food environment and promoting the selection and purchase of unhealthy foods (13, 14).

The daily calorie intake of children at home accounts for about 65%–72% of their total intake, making the family food environment crucial for their dietary intake and nutritional development (15, 16). In Chinese society, there is a strong culture of parental authority, with parents having high control over their children’s diet and limited autonomy for the children (17). Throughout childhood, parents make choices about purchasing and providing food, establish rules regarding meal times, meal frequency, and structure (18, 19). The family’s food consumption determines the types and frequency of food children are exposed to, and the availability and accessibility of healthy food can promote children’s intake of nutritious food (12). If the family provides a variety of fruits, vegetables, and healthy proteins, children have more opportunities to consume a range of nutrients (20, 21).

The nutrition of children is not only related to the family’s dietary consumption, but also to the family’s environmental atmosphere. In Chinese family culture, great importance is placed on family meals, where family members come together to share food, engage in emotional communication, and pass on healthy knowledge to children. This determines that the family environment in Chinese families plays an important role in children’s diet (22–24). A positive dining atmosphere can stimulate children’s appetite, reduce anxiety or resistance towards food, encourage children to taste a variety of foods, balance their nutritional intake, and promote their healthy growth (25, 26).

The theory of the family’s healthy ecosystem suggests that the family is a complex system composed of multiple interactive relationships and roles, with parents being considered as core members who guide and educate children (24, 27–29). Negative emotions of parents can affect children’s nutritional status. If parents display anxious and worried emotions, children will perceive these emotions and experience unease and stress, leading to a decrease in appetite or aversion to food, or using overeating as a way to cope with stress or anxiety (26, 30, 31).

The family is considered the most fundamental and important unit. The COVID-19 pandemic has had a wide-ranging impact on family members’ eating behaviors, childhood overweight and obesity, and intra-family relationships. These aspects have become hot topics in research, but there are still many unknown areas that require further investigation regarding changes in household food consumption as a unit, changes in the Chinese family environment, and the impact of these changes on children’s nutritional status. This study investigated the nutritional status of children after 6 and 18 months of continuous COVID-19 pandemic through a longitudinal study, enriching the understanding of the epidemiological trends of childhood overweight/obesity and malnutrition during the pandemic. Previous research has primarily focused on household dietary consumption during routine periods, but this study examined families as units to complement the understanding of changes in household dietary consumption during the COVID-19 pandemic. In China, family culture emphasizes family cohesion and close relationships, thus this study utilized the Chinese Family Assessment Instrument to assess family atmosphere and explore the relationship between family environment and child nutrition. By analyzing the association between household dietary consumption, family environment, parental anxiety, and child nutrition during the COVID-19 pandemic, this study filled gaps in relevant knowledge and can contribute to the development of effective, family-based interventions during significant public health events.

## 2. Materials and methods

### 2.1. Study design

Chengdu Positive Child Development (CPCD) (32) is a longitudinal cohort study designed to understand the status of children’s positive development as well as psychological, social, and behavioral problems. It also assesses the impact of positive child development programs on promoting the positive development of children. The study employed a multi-stage random cluster sampling method. After stratifying based on geographical and economic conditions, a total of 5 primary and secondary schools in downtown Chengdu, suburban counties, and townships were selected. Students with cognitive disabilities or those unable to independently complete the questionnaire were excluded, and all remaining students were included in the study. With two trained research assistants present in each classroom, data collection was conducted through on-site face-to-face questionnaire interviews in the classroom environment of the selected schools.

This program began with a baseline survey in December 2019. The wave 1 data (W1) was collected in June 2020 and the wave 2 data (W2) was collected in June 2021, with each wave lasting 1 month. This study used data from the first and second rounds, with a sample of 7- to 15-year-old primary and secondary school students and their parents. A total of 7,877 individuals participated in the study. Due to missing matching information between parents and students’ questionnaires, as well as a significant amount of missing data (≥20%) for some questionnaire items, the final sample consisted of 7,645 children aged 7–15.

The study was conducted in accordance with the Helsinki Declaration, and all parents and children participating in the study provided informed consent prior to inclusion. Children also obtained parental informed consent before being included in the study. The research protocol was approved by the Medical.

### 2.2. Measures

A basic information questionnaire was used to collect demographics about the subjects and their parents, including the gender, grade, and age of the elementary school students, and education level, age, and current place of residence.

The nutritional status of children is measured by BMI with reference to the Chinese children’s standard. Due to the restrictions on the aggregation of people during the pandemic, the basic information of students’ height and weight was reported by their parents in both waves of the survey. Overweight, obesity, and malnutrition was defined according to BMI, which was calculated by dividing weight (kg) by the square of height (m) (33, 34). According to the Chinese National Health Commission’s standards, which are based on nationwide surveys of student physical health, this study assessed the nutritional status of Chinese students. These standards take into account the genetic characteristics and environmental influences, such as socioeconomic differences, of the Chinese population. They provide percentile thresholds based on age and gender for screening the nutritional status of school-age children (6–18 years old) in China, including various ethnic groups (35, 36). “Malnutrition” refers to acute malnutrition caused by an immediate deficiency in dietary protein and energy intake, resulting in a body mass index (BMI) below the age-specific BMI threshold range for screening (37, 38). In this study, “moderate to severe malnutrition” and “mild malnutrition” were collectively categorized as “malnutrition.”

Regarding family diet consumption, Parents were asked since June 2020 (the wave 1 survey) about the changes in meal patterns and consumption of major foods during the pandemic, including” going outside to buy groceries,” “using take-out or meal delivery services,” “dine out in the restaurants,” “cooking at home”etc., and check the corresponding box, Including: “decreased,” “decreased a little,” “no change,” “increased a little,” and “increased.” Parents were also asked whether their household consumption of “staple food,” “vegetables,” “fruits,” and “sugary drinks and desserts,” “increased,” “remained the same,” or “decreased” during the COVID-19 pandemic.

Family environment variables were measured using the Chinese Family Assessment Instrument (C-FAI) scale developed by Shek and Ma (39). There were 33 items, and for each item, participants were asked to answer the question, “How similar is this to your family environment?.” Similarity to each statement was scored on a scale of 1–5 out of 165, with higher scores indicating less similarity to the statement. The Cronbach’s ɑ reliability of the scale in this study was tested to be 0.88.

Parental anxiety variables were measured using the Anxiety Self-Assessment Scale (SAS) scale developed by Zung (40), the SAS is an internationally influential anxiety self-assessment tool. It was introduced to China in 1986, but the five items in the scale were difficult for the general public to understand, which affected the accuracy of the scale. Chinese scholars have revised and tested the scale, and achieved a reliability Cronbach’s ɑ of 0.93. The Chinese revised version of the Self-Rating Anxiety Scale has been validated (41, 42). The SAS has 20 items and parents are asked to answer the question “How similar is this to your situation,” each entry is rated on a scale of 1–4 out of 80, the higher the score, the more severe the anxiety, and those with scores of 50 and above are judged to have anxiety. The Cronbach’s ɑ reliability of the scale in this study was tested to be 0.80.

### 2.3. Data analysis

The data was input using EpiData 3.2 and statistical analysis was performed using SPSS 26.0 to calculate the rates among the variables. Chi-square test or T test was used to compare whether the changes among the variables in the two waves of the survey were statistically significant. Multiple logistic regression was used to analyze the effect of changes in family diet consumption on the nutrition status of children and adolescents. A generalized estimation model was used to analyze the effects of family environment and parental anxiety on childhood overweight and obesity. A two-sided test with a test level of 0.05 was used.

## 3. Results

### 3.1. Demographic characteristics

A total of 7,645 students were included in the study, of whom 3,770 (49.31%)were boys and 3,875 (50.69%)were girls; 3,167 (43.43%)were students aged 7–9 years, 3,308 (43.27%)were students aged 10–12 year, and 1,170 (15.30%)were students aged 13–15 years. 5,085 (66.51%)students were living in urban areas and 2,560 (33.49%) students were living in rural areas. 3,368 (44.05%) father’s education level was at junior high school or below, 4,277 (55.95%) at high school and above. 3,471 (45.40%) mother’s education level was at junior high school or below, and 4,714 (54.60%)at high school or above (see Table 1).

**Table 1:** Demographic characteristics.

Variables	Case	Frequency (%)
Gender		
Males	3,770	49.31
Females	3,875	50.69
Age		
7 ~ 9	3,167	41.43
10 ~ 12	3,308	43.27
13 ~ 15	1,170	15.30
Place of residence		
Urban area	5,085	66.51
Rural area	2,560	33.49
Father-education level		
Elementary school	466	6.10
Junior high school	2,902	37.96
Senior high school	2,046	26.76
Vocational school	799	10.45
College or above	1,432	18.73
Mother-education level		
Elementary school	682	8.92
Junior high school	2,789	36.48
Senior high school	1,917	25.08
Vocational school	888	11.62
College or above	1,369	17.91

### 3.2. Nutrition status of children

According to the results, the prevalence rate of malnutrition in wave 1 was 11.64%, the prevalence rate of overweight was 13.11%, and the prevalence rate of obesity was 11.60%. In wave 2, the prevalence rate of malnutrition was 4.96%, the prevalence rate of overweight was 13.73%, and the prevalence rate of obesity was 10.60%. The prevalence rates of malnutrition and obesity in wave1 were higher than those in wave 2, and the differences were statistically significant (*p* < 0.05). As the duration of the COVID-19 pandemic increases, the prevalence of malnutrition and obesity among children has decreased (see Table 2).

**Table 2:** Children’s nutrition, home environment, and parental anxiety in two waves of surveys.

Variables	Total	Malnutrition (%)	Normal (%)	Overweight (%)	Obese (%)
W1^a^ Nutrition	7,645	890 (11.64)	4,866 (63.65)	1,002 (13.11)	887 (11.60)
W2^b^ Nutrition	7,645	379 (4.96)	5,406 (70.71)	1,050 (13.73)	810 (10.60)
*p*	/	*p* < 0.05	*p* < 0.05	*p* > 0.05	*p* < 0.05
W1 FE	1.91 ± 0.72	1.90 ± 0.72	1.89 ± 0.71	1.95 ± 0.74	1.96 ± 0.77
W2 FE	1.87 ± 0.72	1.94 ± 0.74	1.86 ± 0.72	1.84 ± 0.71	1.90 ± 0.72
*p*	*p* < 0.05	*p* < 0.05	*p* < 0.05	*p* < 0.05	*p* < 0.05
W1 PA	476 (6.23)	69 (14.50)	298 (62.61)	58 (12.18)	51 (10.71)
W2 PA	362 (4.74)	25 (6.90)	243 (67.13)	49 (13.54)	45 (12.43)
*p*	*p* < 0.05	*p* < 0.05	*p* < 0.05	*p* < 0.05	*p* < 0.05

FE, Family Environment; PA, Parental Anxiety.

a W1 means wave 1 survey.

b W2 means wave 2 survey.

Overall, the degree of family environmental barriers and the prevalence of parental anxiety were higher in wave 1 than wave 2. The degree of family environmental barriers in malnutrition children and the prevalence rate of parental anxiety in obese children were higher in wave1 than wave 2, and the differences were statistically significant. With the increasing duration of the COVID-19 pandemic, the degree of family environmental barriers increased in malnutrition children, and the level of parental anxiety increased in obese children. The family environment of obese children and the prevalence of parental anxiety in malnutrition children were higher in wave 1 than wave 2, and the differences were statistically significant (*p* < 0.05). With the increasing duration of the COVID-19 pandemic, the degree of family environmental barriers decreased in obese children, and the level of parental anxiety decreased in malnutrition children (see Table 2).

### 3.3. Family diet consumption

Compared to the wave 1 of surveys the wave 2 of surveys 45.49% of households decreased going to the street to buy groceries, 70.15% of households decreased eating behavior at restaurants, 64.89% of households decreased behavior through take-out or meal delivery services, 54.92% of households increased cooking behavior at home. In the second survey, 22.26% of households reduced their consumption of staple foods, 33.23% reduced their consumption of vegetables, 32.28% reduced their consumption of fruits, and 50.70% increase their consumption of sugary beverages and desserts (see Table 3).

**Table 3:** Family diet consumption during the COVID-19 pandemic.

Variables	Decrease	Slightly decrease	Remain unchanged	Slightly increase	Increase
Family eating style					
Going out to buy groceries	3,478 (45.49)	2,561 (33.50)	1,373 (17.96)	158 (2.07)	75 (1.00)
Dining out in the restaurants	5,363 (70.15)	1,478 (19.33)	703 (9.20)	55 (0.72)	46 (0.60)
Take-out (food delivery service)	4,961 (64.89)	1,198 (15.67)	1,045 (13.67)	300 (3.92)	141 (1.84)
Cooking at home	485 (5.99)	182 (2.38)	1,576 (20.61)	1,203 (15.74)	4,199 (54.92)
Main food consumption					
Staple food	1,702 (22.26)	–	5,396 (70.58)	–	547 (7.16)
Vegetable	2,541 (33.23)	–	4,709 (61.60)	–	395 (5.17)
Fruit	2,468 (32.28)	–	4,424 (57.87)	–	753 (9.85)
Sugary drink and dessert	606 (7.92)	–	3,406 (44.55)	–	3,633 (47.52)

### 3.4. Impact of dietary changes on the nutrition status of children

A multifactorial logistic analysis of the wave 2 of the survey showed that the prevalence rate of child malnutrition was higher in households with reduced staple food consumption compared to households with constant staple food consumption (OR = 1.88, 95%CI: 1.05–3.37), higher rates of overweight prevalence among children in families with increased take-out and meal delivery behavior (OR = 1.33, 95%CI: 1.02–1.75) compared to families with constant consumption of take-out and meal delivery services. Families with increased consumption of sugary drinks had higher prevalence rates of childhood obesity compared to families with unchanged sugary drink consumption (OR = 1.22, 95%CI: 1.01–1.48) (see Table 4).

**Table 4:** Multifactorial unordered multi-categorial logistic regression analysis of factors influencing children’s nutrition status.

Nutrition	Variables*	Categories	OR	95%CI	*p*
Malnutrition	Staple food	Decrease	1.88	1.05–3.37	0.035
		Increase	1.52	0.87–2.67	0.141
		Remain unchanged	–	–	–
Overweight	Take-out (food delivery service)	Decrease	1.13	0.62–2.06	0.692
		Slightly decrease	1.14	0.84–1.55	0.407
		Slightly increase	1.15	0.76–1.76	0.508
		Increase	1.33	1.02–1.75	0.036
		Remain unchanged	–	–	–
Obesity	Dietary drink or dessert	Decrease	1.29	0.92–1.80	0.141
		Increase	1.22	1.01–1.48	0.040
		Remain unchanged	–	–	–

^*^Show only variables with meaningful results.

### 3.5. Impact of family environment and parental anxiety on the nutrition status of children

We used GEE models, exchangeable work-related matrices, ordered logistic regression, and main effects models to explore the factors influencing childhood obesity. Participants’ demographic data, family environment, and parental anxiety were entered as factors in the GEE model, and the results of the generalized estimating equations are shown in the table. Low maternal education, low age, living in town, high family dysfunction, and parental anxiety were risk factors for obesity (*p* < 0.05) (see Table 5).

**Table 5:** GEE analysis of children’s nutritional status and family environment, parental anxiety.

Variables	*β*	SE	Wald*χ*^2^	OR	95%CI	*p*
Gender						
Males	0.01	0.04	0.00	1.01	0.92–1.09	0.985
Females	0	–	–	1	–	–
Age						
7 ~ 9	0.20	0.06	9.954	1.22	1.08–1.37	0.002
10 ~ 12	0.44	0.06	60.026	1.55	1.39–1.73	0.000
13 ~ 15	0	–	–	1	–	–
Place of residence						
Urban area	−0.16	0.05	11.65	0.86	0.78–0.94	0.001
Rural area	0	–	–	1	–	–
Father-education level						
Elementary school	−0.18	0.11	2.63	0.84	0.67–1.04	0.105
Junior high school	0.053	0.08	0.44	1.05	0.90–1.23	0.508
Senior high school	0.05	0.08	0.45	1.05	0.90–1.23	0.502
Vocational school	0.07	0.09	0.58	1.07	0.90–1.28	0.447
College or above	0	–	–	1	–	–
Mother-education level						
Elementary school	0.22	0.10	4.52	1.25	1.02–1.53	0.034
Junior high school	0.26	0.087	9.75	1.29	1.10–1.51	0.002
Senior high school	0.21	0.08	6.87	1.23	1.05–1.44	0.009
Vocational school	−0.03	0.09	0.12	0.97	0.81–1.16	0.728
College or above	0	–	–	1	–	–
PA						
Non-anxiety	−0.23	0.08	8.317	0.80	0.68–0.93	0.004
Anxiety	0	–	–	1	–	–
FE^a^						
–	−0.06	0.02	5.192	0.95	0.90–0.99	0.023

SE, standard error; OR, odds ratio; FE, Family environment; PA, Parental Anxiety.

a This variable is a continuous variable.

## 4. Discussion

The aim of this study was to investigate the nutritional status of children in China during the COVID-19 pandemic and its association with family dietary consumption, family environment, and parental anxiety. The findings revealed that increased consumption of unhealthy food by families, chaotic or dysfunctional family environments, and heightened parental psychological stress were risk factors for childhood overweight and obesity. Furthermore, reduced consumption of staple foods by families was identified as a risk factor for childhood malnutrition.

Research has found that during the initial outbreak of the COVID-19 pandemic, the rates of childhood overweight, malnutrition, parental anxiety, and levels of family environmental disruption were higher compared to the period when the pandemic was prevalent. The drastic changes in the environment during the early stages of the COVID-19 outbreak disrupted many systems that children and parents rely on for health, livelihood, education, diet, and exercise (43, 44). The pandemic had an extremely disruptive effect on the family environment, known as the “hammer effect” (45). As the pandemic continued, on one hand, people gradually restored their daily routines, reducing the impact on children’s diet and exercise. On the other hand, families exhibited a certain level of resilience in the face of traumatic stress, which alleviated the psychological distress experienced by family members (46–48). Both family functioning and parental anxiety levels showed signs of recovery.

During the COVID-19 pandemic, reducing staple food consumption in households is a risk factor for child undernutrition. Staple foods are the main source of energy intake. A cohort study has shown that government lockdown measures significantly altered food intake (49). In 2020, the global prevalence of food insufficiency was 8.4%, with 418 million people in Asia unable to access adequate food (4, 50). Due to the severe economic recession caused by the pandemic, food access is jeopardized, and there is a positive correlation between food shortages and malnutrition (51).

Increasing consumption of take-out meals, sugary beverages, and desserts in households is a risk factor for child overweight and obesity. Take-out meals tend to be more energy-dense and have poorer nutrition, often containing high amounts of unhealthy ingredients such as fat, salt, and sugar, which are associated with weight gain and various negative health outcomes (52, 53). Compared to solid foods, sugary beverages provide less satiety and are easier to consume in excess, leading to energy accumulation. On the other hand, sugary beverages have a pleasant taste that can stimulate children’s appetite, leading to increased energy intake (10, 54, 55).

Our research found that family dysfunction and parental anxiety are risk factors for childhood obesity. Epidemiological studies have shown that family functioning plays a significant role in children’s diet and weight status (25). A chaotic family environment exacerbates negative parenting behaviors, leading to children’s preference for high-sugar, high-fat foods (14, 56). Anxious parents may use controlling or restrictive feeding practices, resulting in disrupted eating patterns and emotional eating in children, or they may be more inclined to use food as a means to soothe their children, thereby providing them with more convenient and unhealthy food options (57–59). Previous studies have indicated that there is a bidirectional relationship between family functioning and parental anxiety (60–62). Although this research provides evidence for the relationship between family functioning and nutrition, and parental anxiety and nutrition, the complex mechanisms underlying the interactions between parental anxiety, family functioning, and child nutrition have not been fully elucidated. The extent to which parental anxiety and family functioning jointly contribute to these effects, as well as whether there are mediating or moderating factors between them, requires further research.

## 5. Theoretical implications

Firstly, gaining a better understanding of the nutritional status of children during the ongoing COVID-19 pandemic has highlighted the changes in childhood obesity and malnutrition. This prompts us to not only focus on the issue of childhood obesity during major public health events but also pay attention to the problem of childhood malnutrition. This is important for informing the development of public health policies, which may involve food safety regulations, dietary education, social support systems, and other aspects, in order to protect the nutritional health of children.

This study provides limited evidence on the relationship between changes in household dietary consumption, family environment, parental anxiety, and child nutrition. The study suggests that dietary rules and food accessibility within the family, as well as parental emotions and parenting behaviors, can influence children’s dietary choices and quality. By further applying the theory of the family health ecosystem to research on family nutrition, the study enriches the theoretical understanding in this field. Additionally, it provides a theoretical basis for further analyzing and addressing child nutrition issues. However, it is important to note that the evidence is still limited, and further research is needed to validate and gain a deeper understanding of these relationships. Lastly, exploring the importance of the social environment in child nutrition has significant implications for policymakers and the design of intervention measures.

## 6. Practice implications

Research promotes the development of intervention measures, and family diet can influence children’s eating habits and intake. Family-based dietary interventions may be effective, as families can increase the consumption of healthy foods and reduce the consumption of obesity-promoting foods such as sugary beverages. At the same time, parents can improve their children’s nutritional status by establishing a supportive family environment, improving their own mental well-being, and maintaining family stability. These factors are crucial for promoting healthy eating and overall growth in children.

Research promotes family nutrition education, and the influence of family factors on child nutrition is undoubtedly important. The family is a microcosm of society, and China has its unique cultural background. For example, parents express love through food, and grandparents tend to overfeed children. When intervening in child nutrition, cultural differences should be taken into account (63). Chinese culture emphasizes parental authority and focuses on collectivism (64). In the context of Chinese social culture, it may be very effective to establish children’s healthy behaviors through role modeling and providing healthy diets. In Chinese families, the choice of what to eat is rarely an individual decision. It reminds promoters of healthy eating that nutrition education may be more effective if designed for the entire family rather than just individuals.

The research findings can provide recommendations for governments and relevant organizations to take measures to mitigate the negative impact of the COVID-19 pandemic on children’s nutrition status. This may include providing economic assistance, improving the food supply chain, promoting healthy eating campaigns, and implementing other measures to ensure children have access to adequate nutrition during the pandemic.

## 7. Strength, limitations, and future studies

In this study, a large sample of children and adolescents was used, focusing not only on overweight and obesity, but also on malnutrition. The study focused on the impact of “family-based” eating behaviors on children and adolescents, which was conducted 2 years into the COVID-19 pandemic, helping to complement the impact of families on the nutritional status of children and adolescents in the ongoing pandemic.

However, there are some limitations to the research. Firstly, the study sample was limited to the Sichuan province, which may reduce the generalizability of the findings. Future research could consider recruiting participants from other regions in China to have a more comprehensive understanding of the nutritional status and weight-related behaviors of Chinese children. Secondly, due to the restrictions of the COVID-19 pandemic, the height and weight of children were reported by parents rather than measured by professionals, which may affect the accuracy of the measurements. Future research could consider using professional measurements of height and weight to improve the accuracy of the data. Thirdly, the survey on household food consumption was relatively simple and may not cover the comprehensive aspects of dietary consumption. Future research could design more detailed and scientific questionnaires to have a more comprehensive understanding of household dietary consumption patterns and changes in the consumption of food types. Lastly, there may be other confounding factors that could influence the associations found in the study. Future research could consider other confounding factors such as genetics, seasonality, weather, physical conditions, and lifestyle factors to enhance the reliability of the research findings.

## 8. Conclusion

In summary, the material environment of the family, the emotions of family members, and children’s perceptions of the family environment can all influence children’s eating behaviors and nutritional intake. Research findings indicate that the prevalence of malnutrition and obesity among children may be more severe during the COVID-19 pandemic. As the duration of the pandemic progresses, the rate of malnutrition in children decreases significantly, while the rate of obesity slightly decreases and the rate of overweight slightly increases. Therefore, the issue of childhood overweight and obesity should not be overlooked. The COVID-19 pandemic has also led to changes in family food consumption, family functioning, and parental emotions. Reduced consumption of staple foods by families is a risk factor for child under-nutrition, while increased consumption of take-out food and sugary beverages is a risk factor for child overweight and obesity. Family dysfunction and parental anxiety are also risk factors for childhood obesity. Family food consumption, family environment, and parental emotions are all factors that influence children’s nutrition. This study provides additional insights into the changing trends of childhood overweight, malnutrition, and other related issues during the ongoing COVID-19 pandemic. It also provides a theoretical basis for family-based intervention measures.

## Data availability statement

The raw data supporting the conclusions of this article will be made available by the authors, without undue reservation.

## Ethics statement

The studies involving human participants were reviewed and approved by the Medical Ethics Committee of Sichuan University ethics number: K2020025. Written informed consent to participate in this study was provided by the participants’ legal guardian/next of kin.

## Author contributions

LZ: funding acquisition. LP and RH: methodology. LZ and LJ: project administration and resources. LP: software and writing—original draft. RH, YF, WS, LZ, and LJ: supervision. LP, RH, YF, and WS: writing—review and editing. All authors contributed to the article and approved the submitted version.

## Funding

This work was supported by National Natural Science Foundation of China, Approval No.: 82273748.

## Conflict of interest

The authors declare that the research was conducted in the absence of any commercial or financial relationships that could be construed as a potential conflict of interest.

## Publisher’s note

All claims expressed in this article are solely those of the authors and do not necessarily represent those of their affiliated organizations, or those of the publisher, the editors and the reviewers. Any product that may be evaluated in this article, or claim that may be made by its manufacturer, is not guaranteed or endorsed by the publisher.
