# Supplementary material for: The relationship between family diet consumption, family environment, parent anxiety and nutrition status children during the COVID-19 pandemic: a longitudinal study
Source: Front Public Health. 2023 Aug 10;11:1228626. doi: 10.3389/fpubh.2023.1228626 (PMC10447892; doi:10.3389/fpubh.2023.1228626)
Supplement: Supplementary file 1 [file Data_Sheet_1.PDF]

# The Chinese weight status assessment criteria for children aged 6-18 years.

The assessment criteria for weight status in school-age children (malnutrition, normal weight, overweight, obesity)， The unit "kg/m2".

| Age | Male | | | | | Female | | | | |
|---|---|---|---|---|---|---|---|---|---|---|
| | Severe malnutrition | Mild malnutrition | normal weight | overweight | obesity | Severe malnutrition | Mild malnutrition | normal weight | overweight | obesity |
| 6.0~ | ≤13.2 | 13.3-13.4 | 13.4-16.4 | 16.4-17.7 | >17.7 | ≤12.8 | 12.9-13.1 | 13.1-16.2 | 16.2-17.5 | >17.5 |
| 6.5~ | ≤13.4 | 13.5-13.8 | 13.8-16.7 | 16.7-18.1 | >18.1 | ≤12.9 | 13.0-13.3 | 13.3-16.5 | 16.5-18.0 | >18.0 |
| 7.0~ | ≤13.5 | 13.6-13.9 | 13.9-17.0 | 17.0-18.7 | >18.7 | ≤13.0 | 13.1-13.4 | 13.4-16.8 | 16.8-18.5 | >18.5 |
| 7.5~ | ≤13.5 | 13.6-13.9 | 13.9-17.4 | 17.4-19.2 | >19.2 | ≤13.0 | 13.1-13.5 | 13.5-17.2 | 17.2-19.0 | >19.0 |
| 8.0~ | ≤13.6 | 13.7-14.0 | 14.0-17.8 | 17.8-19.7 | >19.7 | ≤13.1 | 13.2-13.6 | 13.6-17.6 | 17.6-19.4 | >19.4 |
| 8.5~ | ≤13.6 | 13.7-14.0 | 14.0-18.1 | 18.1-20.3 | >20.3 | ≤1 3.1 | 13.2-13.7 | 13.7-18.1 | 18.1-19.9 | >19.9 |
| 9.0~ | ≤13.7 | 13.8-14.1 | 14.1-18.5 | 18.5-20.8 | >20.8 | ≤13.2 | 13.3-13.8 | 13.8-18.5 | 18.5-20.4 | >20.4 |
| 9.5~ | ≤13.8 | 13.9-14.2 | 14.2-18.9 | 18.9-21.4 | >21.4 | ≤13.2 | 13.3-13.9 | 13.9-19.0 | 19.0-21.0 | >21.0 |
| 10.0~ | ≤13.9 | 14.0-14.4 | 14.4-19.2 | 19.2-21.9 | >21.9 | ≤13.3 | 13.4-14.0 | 14.0-19.5 | 19.5-21.5 | >21.5 |
| 10.5~ | ≤14.0 | 14.1-14.6 | 14.6-19.6 | 19 6-22.5 | >22.5 | ≤13.4 | 13.5-14.1 | 14.1-20.0 | 20.0-22.1 | >22.1 |
| 11.0~ | ≤14.2 | 14.3-14.9 | 14.9-19.9 | 19.9-23.0 | >23.0 | ≤13.7 | 13.8-14.3 | 14.3-20.5 | 20.5-22.7 | >22.7 |
| 11.5~ | ≤14.3 | 14.4-15.1 | 15.1-20.3 | 20.3-23.6 | >23.6 | ≤13.9 | 14.0-14.5 | 14.5-21.1 | 21.1-23.3 | >23.3 |
| 12.0~ | ≤14.4 | 14.5-15.4 | 15.4-20.7 | 20.7-24.1 | >24.1 | ≤14.1 | 14.2-14.7 | 14.7-21.5 | 21.5-23.9 | >23.9 |
| 12.5~ | ≤14.5 | 14.6-15.6 | 15.6-21.0 | 21.0-24.7 | >24.7 | ≤14.3 | 14.4-14.9 | 14.9-2 1.9 | 21.9-24.5 | >24.5 |
| 13.0~ | ≤14.8 | 14.9-15.9 | 15.9-21.4 | 21.4-25.2 | >25.2 | ≤14.6 | 14.7-15.3 | 15.3-22.2 | 22.2-24.5 | >25.0 |
| 13.5~ | ≤15.0 | 15.1-16.1 | 16.1-21.9 | 21.9-25.7 | >25.7 | ≤14.9 | 15.0-15.6 | 15.6-22.6 | 22.6-25.6 | >25.6 |
| 14.0~ | ≤15.3 | 15.4-16.4 | 16.4-22.3 | 22.3-26.1 | >26.1 | ≤15.3 | 15.4-16.0 | 16.0-22.8 | 22.8-25.9 | >25.9 |
| 14.5~ | ≤15.5 | 15.6-16.7 | 16.7-22.6 | 22.6-26.4 | >26.4 | ≤15.7 | 15.8-16.3 | 16.3-23.0 | 23.0-26.3 | >26.3 |
| 15.0~ | ≤15.8 | 15.9-16.9 | 16.9-22.9 | 22.9-26.6 | >26.6 | ≤16.0 | 16.1-16.6 | 16.6-23.2 | 23.2-26.6 | >26.6 |
| 15.5~ | ≤16.0 | 16.1-17.0 | 17.0-23.1 | 23.1-26.9 | >26.9 | ≤16.2 | 16.3-16.8 | 16.8-23.4 | 23.4-26.9 | >26.9 |
| 16.0~ | ≤16.2 | 16.3-17.3 | 17.3-23.3 | 23.3-27.1 | >27.1 | ≤16.4 | 16.5-17.0 | 17.0-23.6 | 23.6-27.1 | >27.1 |
| 16.5~ | ≤16.4 | 16.5-17.5 | 17.5-23.5 | 23.5-27.4 | >27.4 | ≤16.5 | 16.6-17.1 | 17.1-23.7 | 23.7-27.4 | >27.4 |
| 17.0~ | ≤16.6 | 16.7-17.7 | 17.7-23.7 | 23.7-27.6 | >27.6 | ≤16.6 | 16.7-17.2 | 17.2-23.8 | 23.8-27.6 | >27.6 |
| 17.5~ | ≤16.8 | 16.9-17.9 | 17.9-23.8 | 23.8-27.8 | >27.8 | ≤16.7 | 16.8-17.3 | 17.3-23.9 | 23.9-27.8 | >27.8 |
| ~18.0 | | | | 24.0-28.0 | >28.0 | | | | 24.0-28.0 | >28.0 |
